# Transcriptome profiles revealed the mechanisms underlying the adaptation of yak to high-altitude environments

**DOI:** 10.1038/s41598-019-43773-8

**Published:** 2019-05-17

**Authors:** Jin-Wei Xin, Zhi-Xin Chai, Cheng-Fu Zhang, Qiang Zhang, Yong Zhu, Han-Wen Cao, Qiu-Mei Ji, Jin-Cheng Zhong

**Affiliations:** 1 State Key Laboratory of Hulless Barley and Yak Germplasm Resources and Genetic Improvement, Lhasa, P. R. China; 2 Institute of Animal Science and Veterinary, Tibet Academy of Agricultural and Animal Husbandry Sciences, Lhasa, P. R. China; 3 Key Laboratory of Qinghai-Tibetan Plateau Animal Genetic Resource Reservation and Utilization, Sichuan Province and Ministry of Education, Southwest Minzu University, Chengdu, P. R. China

**Keywords:** Metabolic pathways, Animal physiology

## Abstract

The yak is a valuable species in the Qinghai-Tibet Plateau of China. Nevertheless, the molecular mechanisms underlying its adaptation to high-altitude environments remain largely unknown. In the present study, comparative transcriptome sequencing was performed for lung and gluteus tissues from two species of low-altitude cattle (Sanjiang and Holstein cattle), Tibetan cattle (living at a moderate altitude), and yak (living at a high altitude) and the differentially expressed genes were validated using real-time quantitative PCR. The results showed that CD36 antigen was up-regulated and CD59 antigen was down-regulated in yak in comparison to the other animals, which might promote the development of red blood cells and inhibit the development of lymphocytes in yak. In addition, thrombospondin type 1, coagulation factor 5/8, and fibronectin were all down-regulated, but serpin and alpha 2-macroglobulin (A2M) were up-regulated. These differences would inhibit blood coagulation, thus reducing the risk of pulmonary edema. The expression levels of the calcium-release, potassium, and transient receptor potential channels decreased in yak, minimizing membrane depolarization and the harmful effects of pulmonary edema. Eleven KEGG pathways associated with innate immunity were more activated in yak and Tibetan cattle than in other cattle strains, which should reduce their risk of infection and disease. These changes together might facilitate the adaptation of yak and Tibetan cattle to live in high-altitude habitats.

## Introduction

The Qinghai-Tibet Plateau in China is one of the harshest places for animals to live, with an average altitude higher than 4000 m, an average air temperature below 10 °C, and an oxygen concentration of only 50–60% of normal values. Yaks (*Bos grunniens*) are the only large mammal living in the Qinghai-Tibet Plateau, making it a valuable species for human use, providing meat, milk, and serving agriculture and transportation purposes^1^.

For adaptation to high-altitude environments, the yak has evolved special morphological and physiological mechanisms. These animals have developed relatively larger lungs and hearts^2^ with much longer, wider, and rounder pulmonary artery endothelial cells^3^ than cattle. Their pulmonary vessels are thin and hypoxic pulmonary vasoconstriction is blunted^4^. The tongue of the yak is shorter and the lingual prominence is greater and more developed, with larger and more numerous conical papillae and thicker keratinized epithelium, than is seen in cattle, enabling yaks to consume a wider variety of pasture plant species^5^. Endogenous purine derivative excretion, average daily urinary N (nitrogen) excretion, fasting daily urinary N excretion, and daily glomerular filtration rates were all lower in yak than in cattle, suggesting that they may have developed special regulating mechanisms in kidney and N metabolism^6,7^. These results partially reveal the morphological, metabolic, and physiological mechanisms underlying the adaptation of yak to high-altitude environments.

Further molecular mechanisms underlying yak adaptation have also been reported. The sequencing of the yak genome was finished in 2012^8^, the findings of which served to suggest that enriched processes of “regulation of blood vessel size”, “regulation of angiogenesis”, “heme binding”, “glycerolipid biosynthetic process”, and “electron carrier activity” might contribute to yak adaptation^8^. Afterwards, several investigations at an mRNA level were conducted. By transcriptome sequencing of the yak lung, Lan, *et al*.^9^, revealed that components of the ribosome and mitochondria, particular immune mechanisms, and the cytochrome oxidase category might be enriched in yak. Transcriptome comparisons between the lung, heart, liver, and kidney of cattle and yak showed that blood supply system, modulation of cardiac contractility, vascular smooth muscle proliferation, and the glutamate receptor system were all likely to be regulated for yak adaptation^10^. Moreover, the microRNA transcriptomes of the heart and lung were compared between yak and cattle, and the subsequent functional analysis revealed that differentially expressed microRNAs were enriched in hypoxia-related pathways, such as the HIF-1α signaling pathway, insulin signaling pathway, PI3K-Akt signaling pathway, nucleotide excision repair, cell cycle, apoptosis, and fatty acid metabolism^2^. All these results are useful in developing the understanding of molecular mechanisms underlying yak adaptation. Nevertheless, these studies only compared transcriptome profiles between yak and one cattle strain. Yak samples were collected from Qinghai-Tibet Plateau, while cattle samples were collected from low-altitude areas. The results of the comparisons between these two species might also indicate short-term stress responses and differences between species, rather than long-time adaptation mechanisms. In the Qinghai-Tibet Plateau, another cattle variety, Tibetan cattle, has been successfully bred to also live in the plateau at an altitude lower than 4500 m. Including Tibetan cattle in a transcriptome analysis might produce more clarity on yak adaptation.

As the most important functional organ in the respiratory system, the lungs are the first organ to react to hypoxic environments^11^. Muscle tissues consume large amounts of oxygen and the metabolism of these two components might be specially regulated in the yak to facilitate their adaptation to high-altitude environments. In the present study, in order to discern the molecular mechanisms underlying yak adaptation, lung and gluteus tissues were collected from Sanjiang cattle (*Bos taurus*, living at low altitude), Tibetan cattle (*B*. *taurus*, living at moderate altitude), Holstein cattle (*Bos taurus*, living at low altitude), and yak (*B*. *mutus*, living at high altitude) for transcriptome sequencing. Bioinformatics analyses were performed to identify differentially expressed genes (DEGs) and enriched pathways. Real-time quantitative PCR was adopted to validate these results. This study will contribute to the knowledge of the molecular mechanisms underlying the adaptation of yak to high-altitude environments.

## Materials and Methods

### Ethics statement

The protocol used in the present study was approved by the Institutional Animal Care and Use Committee, Southwest Minzu University, Chengdu, Sichuan, P. R China. The methods were carried out in accordance with the approved guidelines. During the entirety of the experiments, no local regulations or laws were overlooked. Samples used in the present study were purchased from local farmers.

### Sample collection

Sanjiang cattle (*Bos taurus*), Tibetan cattle, Holstein cattle, and yak (*Bos mutus*) were raised by local farmers. When the animals were sacrificed to obtain meat, fresh gluteus and lung tissues were collected from healthy 60-month old individuals. Samples were immediately frozen in liquid nitrogen. Three replicates from three individuals in each species/strain were collected. Collection date and source location are listed in Table 1.

**Table 1:** Information of samples used in the present study.

Code	Sample name	Sampling location	Coordinates	Altitude	Sampling date
ST	Sanjiang cattle (*Bos taurus*)	Maliu Village, Sanjiang Town, Wenchuan County, Chengdou City	103°22′9.16″E 30°56′36.47″N	1484 m	Oct 17, 2017
TC	Tibetan cattle (*Bos taurus*)	Enda Village, Sangduo Town, Leiwuqi County, Changdou City	96°40′45.72″E 31°7′36.90″N	3791 m	Nov 16, 2017
HC	Holstein cattle (*Bos taurus*)	Taiping Village, Lichun Town, Pengzhou City	103°52′42.43″N 30°58′48.31″E	616 m	Nov 12, 2017
Yak	Yak (*Bos grunniens*)	Keqiong Village, Kamaduo Town, Leiwuqi County, Changdu City	96°22′45.26″N 31°5′54.6″E	4343 m	Nov 6, 2017

For each strain/species, three 90-month old females were collected.

### RNA extraction and transcriptome sequencing

Samples were ground into powder using liquid nitrogen. Total RNA was extracted using Biozol reagent (Bioer, Hangzhou, China), according to the manufacturer’s protocol. The quality of total RNA was monitored on 1% agarose gels and further checked using the NanoPhotometer^®^ spectrophotometer (IMPLEN, CA, USA) and RNA nano 6000 assay kit on Agilent Bioanalyzer 2100 system (Agilent Technologies, CA, USA). An RNA integrity number (RIN) higher than 8.0 was considered qualified. The quantity of RNA was measured using the Qubit^®^ RNA assay kit in Qubit^®^ 2.0 Flurometer (Life Technologies, CA, USA).

In order to construct sequencing libraries, 3 μg of total RNA was treated with the Epicentre Ribo-zeroTM rRNA removal kit (Epicentre, USA) to remove ribosomal RNA and was then harvested by ethanol precipitation. Next, sequencing libraries were prepared using NEBNext^®^ Ultra^TM^ directional RNA library prep kit for Illumina^®^ (NEB, USA). Briefly, RNA was fragmented using divalent cations under elevated temperature in NEBNext first strand synthesis reaction buffer (5X). First strand cDNA was then synthesized using random hexamer primer and M-MuLV reverse transcriptase. Second strand cDNA was synthesized using DNA Polymerase I and RNase H, in which dTTP was replaced by dUTP. The remaining overhangs were removed via exonuclease/polymerase activity. Next, the 3′ ends of the DNA were adenylated and ligated to NEBNext adaptor with a hairpin loop structure for hybridization. The DNA fragments were then purified with AMPure XP system (Beckman Coulter, Beverly, USA) to enrich cDNA fragments of a 250–300 bp length and treated with 3 μl of USER enzyme (NEB, USA), first at 37 °C for 15 min and then at 95 °C for 5 min. DNA fragments were amplified by PCR with Phusion High-Fidelity DNA polymerase, universal PCR primers and index (X) primers. Finally, PCR products were cleaned up using AMPure XP system and library quality was monitored with the Agilent Bioanalyzer 2100 system.

Index-coded samples were clustered on a cBot cluster generation system using a HiSeq. 4000 PE cluster kit (Illumina). Afterwards, DNA libraries were sequenced using an Illumina Hiseq. 4000 platform to collect 150 bp paired-end reads.

### Bioinformatics analyses

After collecting the sequencing data, the adaptors, reads with an N ratio >1%, and low quality reads (with >50% bases having a Phred quality score ≤15) were removed to get clean reads. Clean reads were mapped to the reference genome (BioProject number in GenBank: PRJNA435474) using STAR v2.5.1b^12^. HTseq v0.6.0^13^ was applied to count the numbers of reads mapped to each gene, which were used to calculate the FPKM values (expected number of fragments per kilobase of transcript sequence per million base pairs sequenced). The relative expression levels of each gene among different samples were compared using DESeq2 R package v3.8^14^. Comparisons with a q value < 0.05 were considered statistically significant.

Differentially expressed genes (DEGs) were mapped to the KEGG (Kyoto Encyclopedia of Genes and Genomes) database for enrichment of pathways using clusterProfiler3 v3.8^15^. The significance of KEGG enrichment was corrected to control for the false discovery rate (FDR) using the BH method^16^.

### Real-time quantitative PCR

To validate the expression levels of DEGs produced by Illumina sequencing, qPCR was performed. Seven unigenes with relatively high read counts in one or more species/strains were selected that displayed significant differences among different species/strains. All cDNA was prepared using the BioRT cDNA first strand synthesis kit (Bioer, Hangzhou, China) with oligo(dT) primer. qPCR was performed using BioEasy master mix (Bioer, Hangzhou, China) on a Line Gene9600 Plus qPCR machine (Bioer, Hangzhou, China). Each reaction was repeated three times for technical replicates. The DEGs and primers used for qPCR are listed in Supplementary Table S1. Glyceraldehyde phosphate dehydrogenase (GAPDH) was used as the internal control. The relative expression level of each gene was calculated using the typical 2^−ΔΔCt^ method^17^.

## Results and Discussion

### Illumina sequencing

One lung sample from Sanjiang cattle (SC-2) was unable to be sequenced, due to RNA degradation. Analyses of clean data resulted in 79 M to 101 M of clean reads for lung samples and 75 M to 97 M of clean reads for the gluteus samples. Q20 values were all higher than 96.39% (Supplementary Table S2). These results suggested that the sequencing data were qualified and deep enough for further analyses.

### Differentially expressed genes and qPCR validation

**Figure 1:**
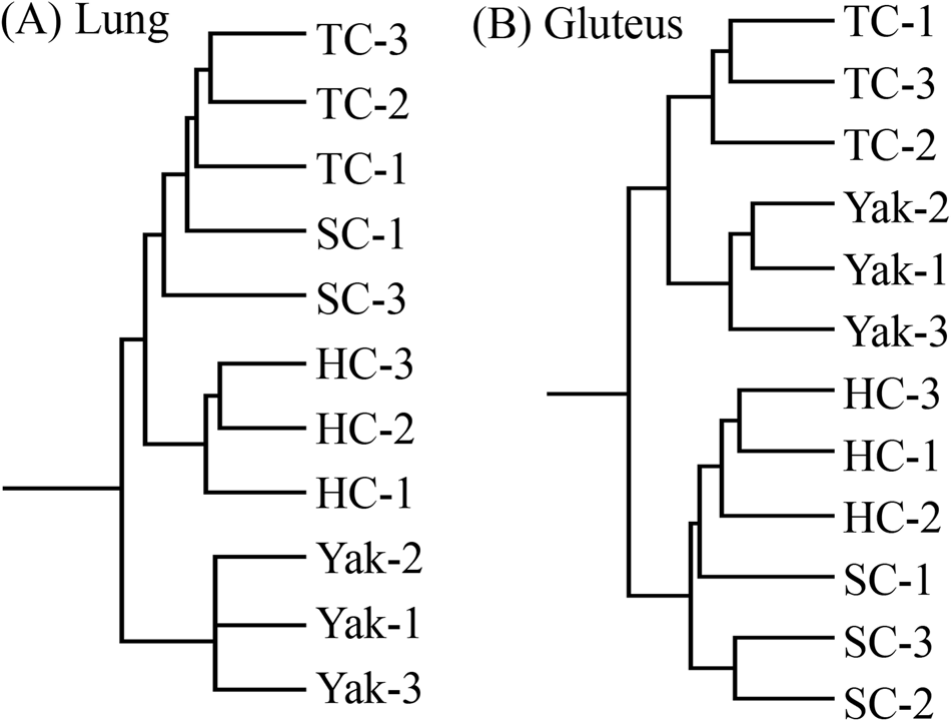
Clustering patterns of transcriptome profiles among yak, Sanjiang cattle (SC), Holstein cattle (HC), and Tibetan cattle (TC). (**A**) lung; (**B**). gluteus.

**Figure 2:**
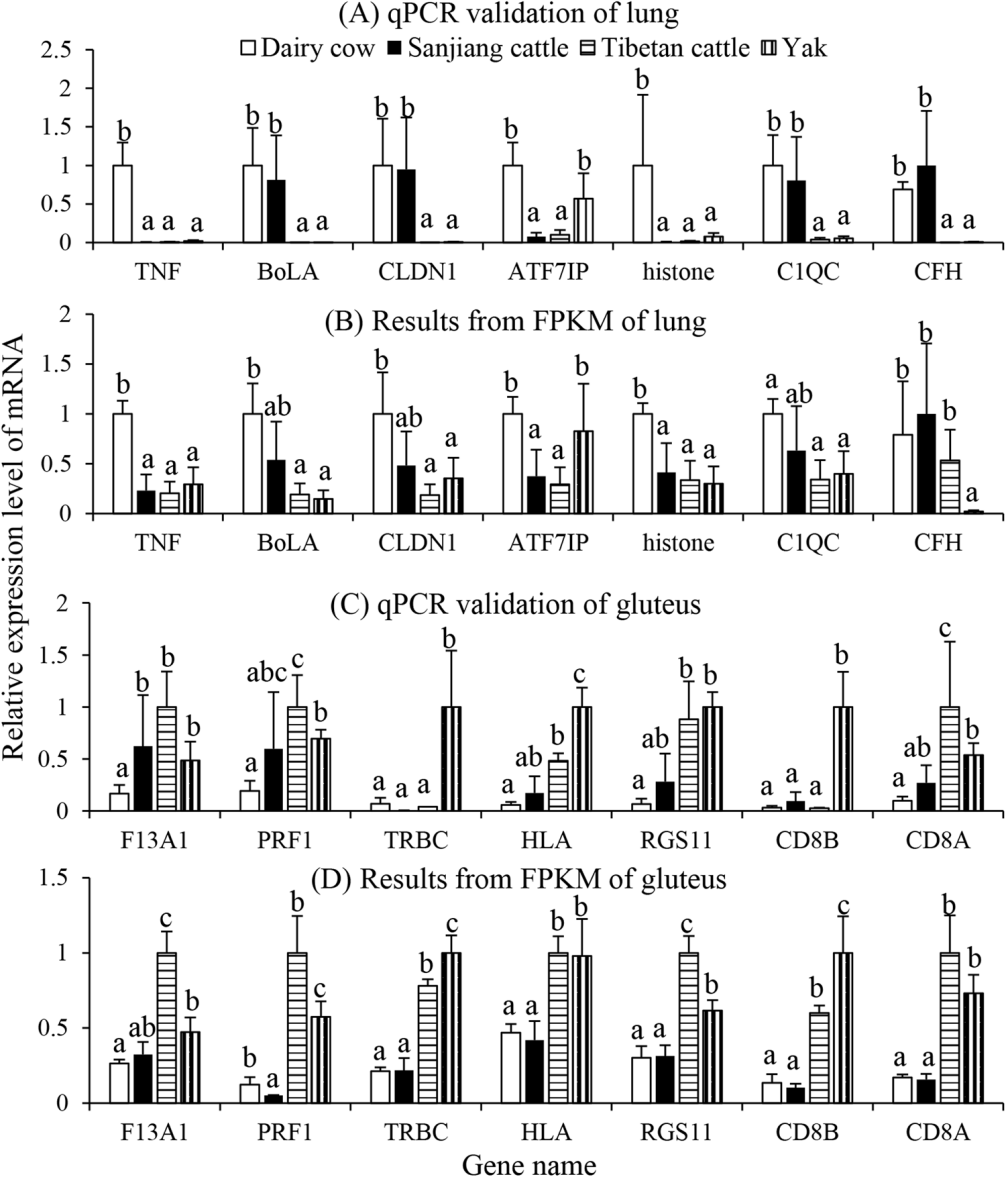
Real-time quantitative PCR validation of differentially expressed genes. Different letters above bars indicate significant differences between variables based on Student’s T-tests (P < 0.05). TNF: tumor necrosis factor; BoLA: Bovine MHC class I; CLDN1: claudin 11; ATF7IP: activating transcription factor 7 interacting protein; C1QC: complement component 1, q subcomponent, C chain; CFH: complement factor H, transcript variant X2; SERPINA1: serpin peptidase inhibitor, clade A; F13A1: coagulation factor XIII, A1 polypeptide; PRF1: perforin 1; TRBC: T-cell receptor beta chain; HLA: human leukocyte antigen gene complex class II histocompatibility antigen; RGS11: regulator of G protein signaling 11; CD8B: cluster of differentiation 8 b molecule; CD8A: cluster of differentiation 8 a molecule.

Pairwise comparisons revealed that hundreds to thousands of genes were differentially expressed among different samples. In order to technically validate these DEGs, seven DEGs in the lung and seven in the gluteus were selected for qPCR (Fig. 1). Overall, the qPCR and FPKM results showed a similar tendency, suggesting the reliability of the transcriptome sequencing results (Fig. 2).

In lung tissue, 1031, 2686, and 799 DEGs existed in yak compared with Sanjiang, Holstein, and Tibetan cattle, respectively. In gluteus tissue, 1545, 1516, and 945 unigenes were significantly differentially expressed in yak in comparison to Sanjiang, Holstein, and Tibetan cattle, respectively (Table 2). Next, H-cluster analysis showed different clustering patterns between lung and gluteus tissues. Based on the results found in lung tissue, yak formed one cluster and Sanjiang, Tibetan, and Holstein cattle formed another. Analyses of the gluteus data displayed two clusters, one including Sanjiang and Holstein cattle, and another containing yak and Tibetan cattle.

**Table 2:** Numbers of differentially expressed genes among samples.

	SC	HC	TC	Yak
SC	—	233	1374	1545
HC	1027	—	1326	1516
TC	239	1761	—	945
Yak	1031	2686	799	—

Blow diagonal: lung tissue; Above diagonal: gluteus. SC: Sanjiang cattle; HC: Holstein cattle; TC: Tibetan cattle.

Tibetan, Holstein, and Sanjiang cattle all belong to *B*. *taurus*, which probably separated from yak (*B*. *mutus*) about 4.4 to 5.3 million years ago^18^. Evolutionarily, yak should be genetically far from Tibetan, Holstein, and Sanjiang cattle, which was consistent with the H-cluster pattern of the lung transcriptome. However, Tibetan cattle and yak live in similar environments. Their adaptation to local environments might regulate mRNA expression in gluteus tissues, and may have finally separated Tibetan cattle from Holstein and Sanjiang cattle on the H-cluster pattern. In addition, Tibetan cattle might obtain significant gene flow from yak^19–21^ and adaptive introgression from yak might also have occurred in Tibetan cattle. A similar phenomenon has been revealed in butterflies^22^ and humans^23^. Thus, local adaptation and introgression might explain the H-cluster results of the gluteus transcriptome. The different clustering patterns suggest that mechanisms underlying the adaption of yak and/or Tibetan cattle to a plateau climate might be different between the lung and gluteus, probably depending on competitive outcome of genetic background and local adaptation. A similar reason could also be used to explain why more DEGs (799) were detected between Tibetan cattle and yak than between Tibetan and Sanjiang cattle (239 DEGs).

### Enrichment of KEGG pathways in the lung and gluteus transcriptome

Comparisons of DEGs in the lung enriched a large number of KEGG pathways. Among them, six pathways were shared between yak and all three cattle strains (Table 3). These pathways were mainly related to the respiratory and circulatory systems, as well as metabolism of signaling molecules.

**Table 3:** Significantly enriched KEGG pathways shared by lung tissue comparisons between yak and Sangjiang/Holstein/Tibetan cattle.

KEGG ID	Name of KEGG pathway	Involved/total gene numbers		
		SC vs Yak	HC vs Yak	TC vs yak
KO04640	Hematopoietic cell lineage	17/419 P = 0.00 Q = 0.00	28/945 P = 0.00 Q = 0.00	14/357 P = 0.00 Q = 0.01
KO00590	Arachidonic acid metabolism	11/419 P = 0.01 Q = 0.10	20/945 P = 0.00 Q = 0.06	13/357 P = 0.00 Q = 0.01
KO04610	Complement and coagulation cascades	10/419 P = 0.00 Q = 0.10	17/945 P = 0.00 Q = 0.07	12/357 P = 0.00 Q = 0.01
KO04020	Calcium signaling pathway	21/419 P = 0.01 Q = 0.11	39/945 P = 0.01 Q = 0.13	19/357 P = 0.01 Q = 0.12
KO04913	Ovarian steroidogenesis	9/419 P = 0.01 Q = 0.10	14/945 P = 0.01 Q = 0.13	7/357 P = 0.02 Q = 0.24
KO05414	Dilated cardiomyopathy	12/419 P = 0.01 Q = 0.11	26/945 P = 0.00 Q = 0.01	9/357 P = 0.04 Q = 0.33

P values indicate statistical significance and Q values represent correction of P values using Benjamini and Hochberg’s method. SC: Sanjiang cattle; HC: Holstein cattle; TC: Tibetan cattle.

Four sets of pairwise comparisons between the gluteus tissues of yak and Tibetan cattle and those of Sanjiang and Holstein cattle displayed 15 shared KEGG pathways (Table 4). According to their functions, these pathways are mainly involved in the respiratory and circulatory systems, immunity process, and cell adhesion and movement.

**Table 4:** Significantly enriched KEGG pathways shared by comparisons between gluteus tissues of yak/Tibetan cattle and Holstein/Sanjiang cattle.

KEGG ID	Name of KEGG pathway	Involved/total gene numbers			
		S vs Y	H vs T	H vs Y	S vs T
ko04650	Natural killer cell mediated cytotoxicity	38/607 P = 0.00 Q = 0.00	41/569 P = 0.00 Q = 0.00	34/632 P = 0.00 Q = 0.00	42/560 P = 0.00 Q = 0.00
ko04612	Antigen processing and presentation	28/607 P = 0.00 Q = 0.00	19/569 P = 0.00 Q = 0.00	17/632 P = 0.01 Q = 0.08	32/560 P = 0.00 Q = 0.00
ko04060	Cytokine-cytokine receptor interaction	46/607 P = 0.00 Q = 0.00	46/569 P = 0.00 Q = 0.00	31/632 P = 0.02 Q = 0.13	49/560 P = 0.00 Q = 0.00
ko04640	Hematopoietic cell lineage	21/607 P = 0.00 Q = 0.00	24/569 P = 0.00 Q = 0.00	21/632 P = 0.00 Q = 0.00	26/560 P = 0.00 Q = 0.00
ko05340	Primary immunodeficiency	14/607 P = 0.00 Q = 0.00	14/569 P = 0.00 Q = 0.00	16/632 P = 0.00 Q = 0.00	20/560 P = 0.00 Q = 0.00
ko04514	Cell adhesion molecules (CAMs)	32/607 P = 0.00 Q = 0.00	23/569 P = 0.01 Q = 0.03	27/632 P = 0.00 Q = 0.02	38/560 P = 0.00 Q = 0.00
ko04145	Phagosome	27/607 P = 0.00 Q = 0.00	24/569 P = 0.00 Q = 0.01	22/632 P = 0.01 Q = 0.10	32/560 P = 0.00 Q = 0.00
ko05416	Viral myocarditis	20/607 P = 0.00 Q = 0.00	17/569 P = 0.00 Q = 0.01	17/632 P = 0.00 Q = 0.06	22/560 P = 0.00 Q = 0.00
ko05330	Allograft rejection	17/607 P = 0.00 Q = 0.00	16/569 P = 0.00 Q = 0.00	12/632 P = 0.04 Q = 0.25	21/560 P = 0.00 Q = 0.00
ko04610	Complement and coagulation cascades	14/607 P = 0.00 Q = 0.01	12/569 P = 0.01 Q = 0.03	12/632 P = 0.01 Q = 0.11	18/560 P = 0.00 Q = 0.00
ko04666	Fc gamma R-mediated phagocytosis	16/607 P = 0.00 Q = 0.02	23/569 P = 0.00 Q = 0.00	14/632 P = 0.00 Q = 0.01	20/560 P = 0.00 Q = 0.00
ko04660	T cell receptor signaling pathway	20/607 P = 0.00 Q = 0.02	23/569 P = 0.00 Q = 0.00	22/632 P = 0.04 Q = 0.26	20/560 P = 0.00 Q = 0.00
ko04662	B cell receptor signaling pathway	11/607 P = 0.03 Q = 0.19	20/569 P = 0.00 Q = 0.00	11/632 P = 0.00 Q = 0.00	19/560 P = 0.00 Q = 0.00
ko04670	Leukocyte transendothelial migration	14/607 P = 0.05 Q = 0.23	17/569 P = 0.00 Q = 0.02	16/632 P = 0.02 Q = 0.12	19/560 P = 0.00 Q = 0.00
ko04810	Regulation of actin cytoskeleton	20/607 P = 0.18 Q = 0.53	27/569 P = 0.00 Q = 0.02	24/632 P = 0.05 Q = 0.27	23/560 P = 0.02 Q = 0.01

P values indicate statistical significance and Q values represent correction of P values using Benjamini and Hochberg’s method. S: Sanjiang cattle; H: Holstein cattle; T: Tibetan cattle; Y: yak.

### Mechanisms underlying regulation of blood cell development in yak lung

High altitude and hypoxia can induce polycythemia. Exposure to high altitudes increases the numbers of red blood cell and platelets, but decreases the numbers of granulocyte/monocyte progenitors (GMPs)^24,25^. In the present study, similar results were revealed. The KEGG pathway hematopoietic cell lineage (KO04640) was enriched in the lung transcriptome between yak and three cattle varieties (Supplementary Table S3). Compared with Sanjiang, Holstein, and Tibetan cattle, interleukin-6 and the interleukin-6 receptor, which are mainly secreted by the function in lymphocytes, were down-regulated in the yak lung, suggesting that the number of lymphocytes might be reduced.

Regarding the regulatory mechanisms underlying blood cell development, CD36 antigen modulates the effects of cell growth factors on the differentiation of erythroid progenitors^26^ and CD59 antigen (LY-6 antigen) is involved in T cell development^27^. In the present study, CD36 antigen was found to be up-regulated in yak, probably increasing the number of red blood cells and down-regulating CD59 antigen in yak, which might be a reason for the decreased proportion of lymphocytes.

### Mechanisms underlying resistance of pulmonary edema in yak

High altitude conditions probably trigger pulmonary edema in animals, which can seriously endanger animal health. Pulmonary edema results from coagulation activation and fibrinolysis inhibition^28–30^. To adapt to high altitude conditions, yaks likely developed mechanisms to prevent such pulmonary abnormalities. In the present study, the expression levels of thrombospondin type 1, coagulation factor 5/8, and fibronectin decreased, but the levels of serpin and alpha 2-macroglobulin (A2M) increased in yak, compared with Tibetan, Holstein, and Sanjiang cattle (Supplementary Table S3). Thrombospondin type 1 functions in blood coagulation and coagulation factor 8 mediates the cross-linking of fibronectin to collagen^31^. Decreased levels of these genes could suppress coagulation. Serpin is also an inhibitor of coagulation^32^ and A2M has an inhibitory activity of human blood coagulation factor Xa^33^. These changes together might decrease blood coagulation and thus reduce the risk of pulmonary edema.

In addition, pulmonary edema depolarizes cell membrane potential and increases the level of cytosolic Ca^2+^ ^34^. In yak, genes involved in membrane depolarization and Ca^2+^ balance were mediated, which might contribute to the resistance to pulmonary edema. The intracellular calcium-release channel (ICRC) mediates the release of Ca^2+^ from extracellular components into the cytoplasm^35^. The voltage-gated potassium channel (VGPC) regulates transmembrane potassium transportation in excitable membranes. Allowing potassium ions to pass in accordance with their electrochemical gradient could minimize membrane depolarization^36^. The transient receptor potential channel, canonical 3 (TRPC-3), is a calcium-activated non-selective cation channel, which could increase the level of intracellular Ca^2+^ during membrane depolarization^37,38^. Compared with other animals, the expression levels of ICRC, VGPC, and TRPC-3 were decreased in yak, which should minimize membrane depolarization caused by high altitude (Supplementary Table S3).

### Potential roles of arachidonic acid metabolism and ovarian steroidogenesis in yak adaptation

As previously reported, fluctuations in the levels of ovarian steroid hormone^39^ and arachidonic acid^40^ affected altitude acclimatization. In the present study, lung transcriptome comparisons between yak and other animals enriched the KEGG pathways arachidonic acid metabolism (KO00590) and ovarian steroidogenesis (KO04913). Within these pathways, aldo-keto reductase catalyzes the conversion of 15KD-PGF_2α_ (prostaglandin F 2α) to 13,14H_2_-PGF_2α_^41^, as well as the conversion of progesterone to 20 alpha-hydroxyprogesterone^42^. Cytochrome P450 CYP2J2 is one of the enzymes responsible for epoxidation of endogenous arachidonic acid pools^43^. Epidermal growth factor could stimulate the release of arachidonic acid^44^ and lipoxygenase could metabolize arachidonic acid^45^. These genes were all up-regulated in yak, suggesting that levels of steroid hormones and arachidonic acid should be relatively higher in yak (Supplementary Table S3). To the best of our knowledge, the biological functions of elevated ovarian steroid hormone and arachidonic acid levels have not been illustrated. One possibility is that, when muscle tissues suffer from excess lactic acid under hypoxic conditions, the activation of arachidonic acid metabolism and ovarian steroidogenesis could promote the repair and growth of the tissues via Akt/mTOR pathway signaling^46^. Alternatively, these hormones might increase myogenic tone and regulate blood flow to resist high-altitude environments^47^.

### Immune system activation in yak gluteus tissues

As reviewed by Mishra and Ganju^48^, high altitude environmental factors, such as UV exposure, cold and hypobaric hypoxia can affect the immune system and make it more susceptible to cancer, various infections, and even autoimmune disease. In the present study, comparisons of the gluteus transcriptome between moderate to high-altitude animals (yak and Tibetan cattle) and low-altitude animals (Sanjiang and Holstein cattle) showed 11 enriched KEGG pathways in relation to immunity (KO04650, KO04612, KO04060, KO05340, KO04145, KO05416, KO05330, KO04666, KO04660, KO04662, and KO04670), and almost all DEGs in these pathways were up-regulated in yak and Tibetan cattle, compared with Sanjiang and Holstein cattle (Supplementary Table S4), suggesting that the immune system was more activated in yak and Tibetan cattle. These changes would allow resistance to infections and disease, facilitating adaptation to a high-altitude environment.

Moreover, cytokines, including chemokines, interferons, interleukins (IL), lymphokines, and tumor necrosis factors, mediate signaling in the innate immunity system. Upregulation of these unigenes indicated that the cytokine-cytokine receptor interaction was activated in yak and TC, which might initiate other KEGG pathways responsible for innate immunity. Similarly, IL-3 and IL-6, perhaps combined with other altered cytokines, were found to be elevated in hypoxic mice^25^.

## Conclusions

Overall, the adaptation of yaks to a high altitude environment probably occurred through the regulation of genes involved in the proliferation of red blood cells, cell membrane depolarization, increasing levels of arachidonic acid and ovarian steroid, and the activation of innate immunity.

## Supplementary information

Supplementary tables
