# Supplementary material for: Transcriptome profiles revealed the mechanisms underlying the adaptation of yak to high-altitude environments
Source: Sci Rep. 2019 May 17;9:7558. doi: 10.1038/s41598-019-43773-8 (PMC6525198; doi:10.1038/s41598-019-43773-8)
Supplement: Supplementary file 1 — Supplementary tables [file 41598_2019_43773_MOESM1_ESM.docx]

**Transcriptome profiles revealed the mechanisms underlying the adaptation of yak to high-altitude environments**

Jin-Wei Xin^1,2,#^, Zhi-Xin Chai^3,#^, Cheng-Fu Zhang^1,2^, Qiang Zhang^1,2^, Yong Zhu^1,2^, Han-Wen Cao^1,2^, Qiu-Mei Ji^1,2,*^, Jin-Cheng Zhong^3,*^

^1^. State Key Laboratory of Hulless Barley and Yak Germplasm Resources and Genetic Improvement, Lhasa, P. R. China

^2^. Institute of Animal Science and Veterinary, Tibet Academy of Agricultural and Animal Husbandry Sciences, Lhasa, P. R. China

^3^. Key Laboratory of Animal Genetics and Breeding, State Ethnic Affairs Commission and Ministry of Education, Southwest Minzu University, Chengdu, P. R. China.

^#^ J-W Xin and Z-X Chai contributed equally to this work.

^*^ Corresponding author: Q-M Ji ([xinjinwei18@163.com)](mailto:xinjinwei18@163.com)) and J-C Zhong ([zhongjincheng518@126.com](mailto:zhongjincheng518@126.com))

**Supplementary Table S1. Primers used in the present study.**

| Gene name | Direction | Primer sequence (5' to 3') |
| --- | --- | --- |
| coagulation factor XIII, A1 polypeptide (F13A1) | Forward | TGGGGAGGCATCATGTTCAT |
|  | Reverse | GGTTAGCTCGCTCACAGTTG |
| perforin 1 (pore forming protein) (PRF1) | Forward | CTTCAACACCTCCACCAAGC |
|  | Reverse | CTCCTCACATGCCTTGAACG |
| T-cell receptor beta chain (TRBC) | Forward | GGACTTACCCCTCATCGTGG |
|  | Reverse | GGACCCGGCTCAGATCATT |
| human leukocyte antigen gene complex class II histocompatibility antigen (HLA) | Forward | GGACAGAAAAGGTGCGGTTT |
|  | Reverse | GGTTGTACTCTCCTCCCCAC |
| regulator of G protein signaling 11 (RGS11) | Forward | TTCAACAAGCCCTGGGAAGA |
|  | Reverse | TGTGCATCTTGGCCCTCTTA |
| cluster of differentiation 8 b molecule (CD8B) | Forward | GTATGGCAAGGAGGTGGACT |
|  | Reverse | CTGGTCTTCTTGGTTGGCTG |
| cluster of differentiation 8 a molecule (CD8A) | Forward | GAGAAGGTGGAGCTGCAATG |
|  | Reverse | GGGTGAGCTGGAATTTGGTG |
| tumor necrosis factor (TNF)\ | Forward | TAGCCGACATCAACTCTCCG |
|  | Reverse | ATGGTGTGGGTGAGGAACAA |
| MHC class I BoLA gene (BoLA) | Forward | ATCTACAAGGACACCGCACA |
|  | Reverse | GGTAGTTCCTCTCGCTCTCC |
| claudin 11 (CLDN11) | Forward | ATTCTTGTGGCTCTCTGTGC |
|  | Reverse | TGGCATGAGTAGGGGAACTG |
| activating transcription factor 7 interacting protein (ATF7IP) | Forward | AAGGGCTAGGTAAGGGAGGT |
|  | Reverse | AACACTTTGAGCACTCCACG |
| histone H3.1 (histone) | Forward | GCATCTCGTCCAAAGCCATG |
|  | Reverse | ACTGGTGTACTTGGTGACGG |
| complement component 1, q subcomponent, C chain (C1QC) | Forward | AGTCCCTGGCCTCTACTACT |
|  | Reverse | GCCGGAGAAAACACTGTCAG |
| complement factor H, transcript variant X2 (CFH) | Forward | GGTCAGGAGAACAAGTGGCT |
|  | Reverse | AACGTGCTCTCTCTCCACTC |
| serpin peptidase inhibitor, clade A (alpha-1 antiproteinase, antitrypsin) (SERPINA1) | Forward | ACCGAGGTCTTCAGCGATAG |
|  | Reverse | GATTCACCACCTTTCCCACG |
| glyceraldehyde phosphate dehydrogenase (GAPDH) | Forward | CAAGTTCAACGGCACAGTCA |
|  | Reverse | GGTGCAGAGATGATGACCCT |

**Supplementary Table S2. Summary of clean data of transcriptome sequencing.** SC: Sanjiang cattle; HC: Holstein cow; TC: Tibetan cattle.

| Sample name | Total reads | Total bases | GC content | Q20 | Q30 |
| --- | --- | --- | --- | --- | --- |
| Lung | | | | | |
| SC-1 | 79,633,986 | 11,945,097,900 | 46.21% | 96.84% | 92.44% |
| SC-3 | 89,332,418 | 13,399,862,700 | 48.71% | 96.39% | 91.56% |
| HC-1 | 79,441,794 | 11,916,269,100 | 49.65% | 96.85% | 92.07% |
| HC-2 | 85,471,174 | 12,820,676,100 | 52.24% | 96.91% | 92.15% |
| HC-3 | 85,152,226 | 12,772,833,900 | 53.72% | 96.87% | 92.04% |
| TC-1 | 90,393,918 | 13,559,087,700 | 44.83% | 97.31% | 93.36% |
| TC-2 | 92,410,554 | 13,861,583,100 | 47.03% | 97.30% | 93.27% |
| TC-3 | 83,735,100 | 12,560,265,000 | 50.44% | 96.94% | 92.60% |
| Yak-1 | 101,042,550 | 15,156,382,500 | 47.80% | 96.77% | 92.18% |
| Yak-2 | 79,658,978 | 11,948,846,700 | 46.77% | 96.98% | 92.60% |
| Yak-3 | 85,586,970 | 12,838,045,500 | 49.09% | 97.22% | 93.06% |
| Gluteus | | | | | |
| SC-1 | 93,179,084 | 13,976,862,600 | 48.98% | 97.59% | 93.90% |
| SC-2 | 95,281,072 | 14,292,160,800 | 51.59% | 97.39% | 93.45% |
| SC-3 | 96,549,672 | 14,482,450,800 | 49.85% | 97.36% | 93.40% |
| HC-1 | 74,960,610 | 11,244,091,500 | 51.77% | 96.90% | 92.14% |
| HC-2 | 82,324,796 | 12,348,719,400 | 50.24% | 96.95% | 92.27% |
| HC-3 | 88,573,368 | 13,286,005,200 | 53.24% | 96.72% | 91.75% |
| TC-1 | 95,064,446 | 14,259,666,900 | 49.42% | 97.48% | 93.64% |
| TC-2 | 169,211,918 | 25,381,787,700 | 54.21% | 96.26% | 90.94% |
| TC-3 | 86,298,348 | 12,944,752,200 | 50.95% | 97.11% | 92.74% |
| Yak-1 | 87,590,344 | 13,138,551,600 | 50.55% | 97.09% | 92.91% |
| Yak-2 | 82,711,736 | 12,406,760,400 | 49.30% | 97.15% | 92.88% |
| Yak-3 | 80,199,662 | 12,029,949,300 | 52.52% | 96.74% | 91.99% |

**Supplementary Table S3. Expression level of genes in lung of Sanjiang cattle, Tibetan cattle, Holstein cow and yak.** Data show mean ± standard error of FPKM.

| Unigene ID | Description | SC | HC | TC | Yak |
| --- | --- | --- | --- | --- | --- |
| ko04610 Complement and coagulation cascades | | | | | |
| BmuPB010057 | fibronectin | 0.8±0.2 | 1.3±0.1 | 1.1±0.2 | 0.3±0.1 |
| BmuPB006008 | thrombospondin type 1 | 0.7±0.1 | 0.7±0.2 | 0.9±0.1 | 0.2±0.0 |
| BmuPB001916 | Serpin family | 0.1±0.1 | 0.1±0.1 | 0.2±0.1 | 1.0±0.1 |
| BmuPB014538 | Alpha-2-macroglobulin | 11.0±2.6 | 8.3±0.6 | 17.1±1.3 | 21.1±1.3 |
| BmuPB003576 | Coagulation factor 5/8 C-terminal type | 4.7±1.7 | 5.3±1.4 | 5.4±0.9 | 2.2±0.4 |
| ko04640 Hematopoietic cell lineage | | | | | |
| BmuPB008438 | interleukin-6 | 4.5±1.3 | 5.9±2.9 | 4.3±1.1 | 0.8±0.1 |
| BmuPB019694 | interleukin-6 receptor | 62.6±17.0 | 64.5±8.3 | 43.0±3.3 | 0.9±0.2 |
| BmuPB008479 | CD59 antigen | 618.7±117.9 | 1042.5±162.0 | 380.0±103.8 | 292.1±41.3 |
| BmuPB008405 | CD36 antigen | 83.8±18.3 | 67.6±3.8 | 78.0±9.0 | 189.5±11.9 |
| BmuPB008438 | Proenkephalin A | 4.5±1.3 | 5.9±2.9 | 4.3±1.1 | 0.8±0.1 |
| BmuPB007163 | Immunoglobulin subtype 2 | 2.2±1.0 | 3.0±1.8 | 0.8±0.1 | 0.7±0.1 |
| BmuPB015356 | Gap junction alpha-8 protein (Cx50) | 74.1±35.7 | 32.3±1.2 | 55.6±7.7 | 107.3±17.4 |
| BmuPB010057 | GPCR, family 3, metabotropic glutamate receptor 1 | 0.8±0.2 | 1.3±0.1 | 1.1±0.2 | 0.3±0.1 |
| ko04020 Calcium signaling pathway | | | | | |
| BmuPB017510 | Transient receptor potential channel, canonical 3 | 0.0±0.0 | 0.1±0.1 | 0.1±0.1 | 2.1±0.3 |
| BmuPB006008 | Potassium channel, voltage dependent, Kv1.6 | 0.7±0.1 | 0.7±0.2 | 0.9±0.1 | 0.2±0.0 |
| BmuPB011827 | Phospholipase C, phosphatidylinositol-specific, Y domain | 4.1±0.1 | 6.0±0.5 | 4.6±0.4 | 23.0±0.6 |
| BmuPB016474 | Intracellular calcium-release channel | 0.3±0.2 | 0.3±0.1 | 0.5±0.2 | 0.1±0.0 |
| ko00590 Arachidonic acid metabolism | | | | | |
| BmuPB009268 | Epidermal growth factor-like domain | 37.1±4.7 | 61.1±7.1 | 40.5±0.7 | 86.6±2.2 |
| BmuPB000974 | Cytochrome P450, CYP2 family | 323.8±99.1 | 447.1±46.5 | 581.6±62.0 | 900.5±70.6 |
| BmuPB003100 | Lipoxygenase | 0.4±0.0 | 0.3±0.1 | 0.3±0.0 | 1.0±0.1 |
| ko04913 Ovarian steroidogenesis | | | | | |
| BmuPB000784 | Aldo/keto reductase | 3.5±1.4 | 5.5±0.9 | 4.8±1.8 | 22.2±5.2 |
| BmuPB000781 | Aldo/keto reductase | 2.2±0.9 | 2.2±0.3 | 5.4±1.6 | 7.6±0.9 |
| BmuPB000790 | Aldo/keto reductase | 5.9±5.1 | 0.8±0.3 | 0.4±0.1 | 43.2±6.5 |

**Supplementary Table S4. Expression level of genes in gluteus of Sanjiang cattle, Tibetan cattle, Holstein cow and yak.** Data show mean ± standard error of FPKM.

| Unigene ID | Description | SC | HC | TC | Yak |
| --- | --- | --- | --- | --- | --- |
| ko04650 Natural killer cell mediated cytotoxicity | | | | | |
| BmuPB021452 | C-type lectin | 1.0±0.3 | 0.9±0.3 | 6.5±1.0 | 9.3±1.9 |
| BmuPB021446 | C-type lectin | 1.6±0.3 | 1.8±0.7 | 15.0±4.1 | 13.1±1.0 |
| BmuPB021448 | C-type lectin | 2.4±1.0 | 2.8±0.6 | 20.1±0.7 | 16.3±3.0 |
| BmuPB001097 | Haem peroxidase | 1.1±0.4 | 2.3±0.9 | 10.6±0.7 | 12.0±1.8 |
| BmuPB021100 | Blood group Rhesus C/E/D polypeptide | 2.0±1.8 | 3.2±0.7 | 26.5±19.4 | 1.3±0.5 |
| BmuPB021098 | Blood group Rhesus C/E/D polypeptide | 1.4±0.2 | 2.0±0.4 | 14.8±6.5 | 0.9±0.4 |
| BmuPB021357 | MHC class I alpha | 314.2±212.5 | 65.3±16.5 | 650.1±345.9 | 1127.9±84.9 |
| BmuPB020669 | Small GTPase superfamily | 3.2±0.1 | 4.6±1.7 | 17.3±2.3 | 14.6±2.1 |
| BmuPB004468 | Voltage-dependent calcium channel, L-type, beta | 1.6±0.2 | 2.2±0.8 | 9.2±1.4 | 6.2±1.6 |
| ko04612 Antigen processing and presentation | | | | | |
| BmuPB021454 | HMW glutenin | 0.1±0.1 | 0.1±0.1 | 3.4±2.4 | 3.4±0.5 |
| BmuPB007855 | C-type lectin | 0.2±0.1 | 0.2±0.1 | 2.9±1.1 | 2.5±0.9 |
| BmuPB021452 | C-type lectin | 1.0±0.3 | 0.9±0.3 | 6.5±1.0 | 9.3±1.9 |
| BmuPB021446 | C-type lectin | 1.6±0.3 | 1.8±0.7 | 15.0±4.1 | 13.1±1.0 |
| BmuPB017231 | Blood group Rhesus C/E/D polypeptide | 0.5±0.1 | 0.6±0.3 | 2.7±0.2 | 4.6±1.1 |
| BmuPB017232 | Prostaglandin DP receptor | 0.9±0.2 | 1.0±0.1 | 5.9±1.5 | 4.3±0.7 |
| BmuPB021449 | C-type lectin | 0.4±0.1 | 0.9±0.3 | 2.8±0.8 | 3.6±1.3 |
| ko04060 Cytokine-cytokine receptor interaction | | | | | |
| BmuPB002890 | Chemokine interleukin-8 | 0.2±0.1 | 0.5±0.4 | 7.0±2.1 | 1.6±0.3 |
| BmuPB002869 | Chemokine interleukin-8 | 2.2±0.3 | 1.6±0.4 | 28.8±12.8 | 11.7±4.2 |
| BmuPB006567 | Tumour necrosis factor | 0.2±0.1 | 0.6±0.0 | 2.1±0.7 | 3.2±0.9 |
| BmuPB012375 | RNA polymerase sigma factor 54 | 3.7±0.8 | 6.9±5.7 | 46.7±12.5 | 25.1±4.7 |
| BmuPB018032 | Vomeronasal receptor, type 1 | 0.5±0.1 | 0.5±0.2 | 3.2±0.3 | 2.7±0.2 |
| BmuPB015933 | TNFR/NGFR cysteine-rich region | 2.2±0.9 | 1.5±0.3 | 2.3±0.5 | 18.2±4.3 |
| BmuPB020673 | Cadherin | 1.0±0.1 | 1.4±0.4 | 5.0±1.0 | 8.3±1.9 |
| BmuPB002694 | Protein kinase | 0.6±0.1 | 0.6±0.2 | 1.8±0.4 | 4.7±0.7 |
| ko04666 Fc gamma R-mediated phagocytosis | | | | | |
| BmuPB021095 | Blood group Rhesus C/E/D polypeptide | 0.6±0.1 | 0.4±0.2 | 6.9±1.9 | 1.9±0.4 |
| BmuPB021100 | Blood group Rhesus C/E/D polypeptide | 2.0±1.8 | 3.2±0.7 | 26.5±19.4 | 1.3±0.5 |
| BmuPB020669 | Small GTPase superfamily | 3.2±0.1 | 4.6±1.7 | 17.3±2.3 | 14.6±2.1 |
| BmuPB013022 | Cadherin | 2.0±0.4 | 2.7±0.3 | 8.8±1.3 | 9.7±1.1 |
| BmuPB009690 | Cadherin | 1.6±0.2 | 1.2±0.3 | 6.2±1.3 | 4.7±0.8 |
| BmuPB010630 | High mobility group, HMG-I/HMG-Y | 12.1±1.7 | 13.5±3.4 | 53.7±6.2 | 42.1±5.8 |
| BmuPB019478 | PAK-box/P21-Rho-binding | 1.3±0.2 | 1.6±0.2 | 5.2±1.0 | 5.8±0.9 |
| BmuPB009689 | Histamine H3 receptor | 1.8±0.6 | 2.2±0.7 | 8.2±0.2 | 6.5±0.9 |
| ko04660 T cell receptor signaling pathway | | | | | |
| BmuPB017231 | Blood group Rhesus C/E/D polypeptide | 0.5±0.1 | 0.6±0.3 | 2.7±0.2 | 4.6±1.1 |
| BmuPB019596 | Neutrophil cytosol factor 2 p67phox | 1.0±0.1 | 1.2±0.3 | 6.6±0.3 | 7.1±0.7 |
| BmuPB018032 | Vomeronasal receptor, type 1 | 0.5±0.1 | 0.5±0.2 | 3.2±0.3 | 2.7±0.2 |
| BmuPB017232 | Prostaglandin DP receptor | 0.9±0.2 | 1.0±0.1 | 5.9±1.5 | 4.3±0.7 |
| BmuPB012636 | Protein kinase | 2.1±0.3 | 2.6±0.9 | 11.5±1.2 | 9.8±0.7 |
| BmuPB005339 | Blood group Rhesus C/E/D polypeptide | 3.6±1.2 | 4.6±1.1 | 18.1±1.2 | 18.5±1.5 |
| BmuPB005338 | Peptidase A22A, presenilin | 3.8±1.1 | 3.8±1.0 | 19.4±1.2 | 14.7±3.0 |
| ko04662 B cell receptor signaling pathway | | | | | |
| BmuPB020669 | Small GTPase superfamily | 3.2±0.1 | 4.6±1.7 | 17.3±2.3 | 14.6±2.1 |
| BmuPB006610 | Phosphatidylinositol 3-kinase Ras-binding | 1.0±0.2 | 1.1±0.3 | 4.4±1.0 | 3.1±0.4 |
| BmuPB013415 | Protein-tyrosine phosphatase, receptor/non-receptor type | 5.5±0.4 | 5.6±0.7 | 22.8±3.1 | 13.1±1.1 |
| BmuPB009995 | Dbl homology | 1.9±0.1 | 1.8±0.2 | 6.8±1.8 | 4.5±0.4 |
| BmuPB004389 | C2 calcium-dependent membrane targeting | 1.2±0.2 | 1.5±0.3 | 5.3±1.0 | 2.9±0.6 |
| BmuPB007257 | Guanine-nucleotide dissociation stimulator CDC25 | 3.8±0.9 | 4.4±0.5 | 10.8±0.8 | 13.0±0.6 |
| BmuPB005699 | Inositol polyphosphate-related phosphatase | 1.5±0.1 | 1.7±0.7 | 5.3±1.3 | 4.1±1.0 |
| BmuPB006893 | SH2 domain | 0.4±0.1 | 0.7±0.2 | 1.5±0.3 | 1.1±0.1 |
| ko04670 Leukocyte transendothelial migration | | | | | |
| BmuPB019596 | Neutrophil cytosol factor 2 p67phox | 1.0±0.1 | 1.2±0.3 | 6.6±0.3 | 7.1±0.7 |
| BmuPB020669 | Small GTPase superfamily | 3.2±0.1 | 4.6±1.7 | 17.3±2.3 | 14.6±2.1 |
| BmuPB004468 | Voltage-dependent calcium channel, L-type, beta subunit | 1.6±0.2 | 2.2±0.8 | 9.2±1.4 | 6.2±1.6 |
| BmuPB006610 | Phosphatidylinositol 3-kinase Ras-binding | 1.0±0.2 | 1.1±0.3 | 4.4±1.0 | 3.1±0.4 |
| BmuPB000322 | G protein-coupled receptor, rhodopsin | 11.8±3.6 | 7.0±2.3 | 34.4±5.8 | 31.1±5.7 |
| BmuPB010327 | Small GTPase superfamily | 1.3±0.3 | 0.8±0.3 | 3.2±0.5 | 3.9±0.5 |
| BmuPB004501 | von Willebrand factor, type A | 1.6±0.2 | 3.6±1.5 | 13.8±8.0 | 3.9±0.7 |
| BmuPB008029 | Orexin receptor family | 4.5±0.7 | 6.3±2.4 | 22.2±6.5 | 13.9±1.0 |
